# High-Mobility Group Box-1 Protein Serum Levels Do Not Reflect Monocytic Function in Patients with Sepsis-Induced Immunosuppression

**DOI:** 10.1155/2010/745724

**Published:** 2010-06-21

**Authors:** Nadine Unterwalder, Christian Meisel, Konstantinos Savvatis, Ben Hammoud, Christina Fotopoulou, Hans-Dieter Volk, Petra Reinke, Joerg C. Schefold

**Affiliations:** ^1^Department of Medical Immunology, Charité University Medicine, Campus Mitte, 10117 Berlin, Germany; ^2^Berlin-Brandenburg Center for Regenerative Therapies (BCRT), Charité University Medicine, Augustenburger Platz 1, 13353 Berlin, Germany; ^3^Department of Cardiology and Pneumology, Charité University Medicine, Campus Benjamin Franklin, 12203 Berlin, Germany; ^4^Department of Gynecology, Charité University Medicine, Campus Virchow Clinic, 13353 Berlin, Germany; ^5^Department of Nephrology and Intensive Care Medicine, Charité University Medicine, Campus Virchow Clinic, 13353 Berlin, Germany

## Abstract

*Background*. High-mobility group box-1 (HMGB-1) protein is released during “late sepsis” by activated monocytes. We investigated whether systemic HMGB-1 levels are associated with indices of monocytic activation/function in patients with sepsis-induced immunosuppression. *Methodology*. 36 patients (31 male, 64 ± 14 years) with severe sepsis/septic shock and monocytic deactivation (reduced mHLA-DR expression and TNF-*α* release) were assessed in a subanalysis of a placebo-controlled immunostimulatory trial using GM-CSF. HMGB-1 levels were assessed over a 9-day treatment interval. Data were compared to standardized biomarkers of monocytic immunity (mHLA-DR expression, TNF-*α* release). *Principle findings*. HMGB-1 levels were enhanced in sepsis but did not differ between treatment and placebo groups at baseline (14.6 ± 13.5 versus 12.5 ± 11.5 ng/ml, *P* = .62). When compared to controls, HMGB-1 level increased transiently in treated patients at day 5 (27.8 ± 21.7 versus 11.0 ± 14.9, *P* = .01). Between group differences were not noted at any other point of assessment. HMGB-1 levels were not associated with markers of monocytic function or clinical disease severity. *Conclusions*. GM-CSF treatment for sepsis-induced immunosuppression induces a moderate but only transient increase in systemic HMGB-1 levels. HMGB-1 levels should not be used for monitoring of monocytic function in immunostimulatory trials as they do not adequately portray contemporary changes in monocytic immunity.

## 1. Introduction

High-mobility group box-1 (HMGB-1) protein, also referred to as amphoterin, is a highly conserved protein that is constitutively expressed in immune cells including monocyte/macrophages, dendritic cells, and neutrophils. HMGB-1 is known as a nuclear DNA-binding protein that is required for transcriptional regulation and gene expression [1, 2]. 

In sepsis, HMGB-1 is typically released by activated innate immune cells in the later phase of the disease [2–4]. Here, HMGB-1 release occurs in response to a number of “alarm signals” such as endotoxin, interferons and tumor necrosis factors and largely is a consequence of NF*κ*B activation and HMGB-1 acetylation at its nuclear localisation site [5, 6]. This induces vesicular sequestration and leads to extracellular HMGB-1 release [1, 2]. In addition to active secretion by activated monocytes/macrophages, passive diffusion from necrotic cells may occur [1, 7].

Once released into the systemic circulation, receptor binding of HMGB-1 to RAGE and toll-like receptors promotes chemotaxis, activates macrophages to release cytokines (e.g., interleukins, IL)/chemokines, inhibits phagocytosis (e.g., of apoptotic neutrophils), and may facilitate recognition and clearance of bacterial products [3, 4, 8–11]. Clinically, however, this may support development of acute lung injury, vascular leakage, tissue hypoperfusion, and endothelial activation [12, 13]. Targeting of HMGB-1 via, for example, specific neutralising antibodies seems therefore appealing as it was shown in animal models that this may protect rodents from lethal sepsis [14, 15]. Due to a rather “wide” therapeutic window, blockade of HMGB-1 is currently investigated in patients with “late” sepsis.

In addition to therapeutic approaches aiming to block or neutralize specific mediators in sepsis, modulation of cellular immunity in an effort to restore adaptive immune responses was proposed. This was done as today many patients do not die from an overwhelming septic burden in the initial phase of the disease but rather in a state of immunologic anergy from complications including severe secondary/nosocomial infections [16–20]. Previously, we and others could demonstrate that reversal of monocytic deactivation may be achieved using measures of both immunostimulation (e.g., interferon-*γ* or granulocyte-macrophage colony stimulating factor, GM-CSF) [21–23] and extracorporeal removal of inhibitory factors [24]. This may especially be useful in the later stages of sepsis [21–25].

It is currently unknown whether immunostimulatory therapies aiming to restore monocytic function influence systemic HMGB-1 levels. This seems of interest as HMGB-1 release is known to occur from activated monocytes/macrophages and may be a potential side effect of such immunostimulatory therapies. In the present analysis, we aim to investigate the “in vivo interplay” between monocytic function (assessed using monocytic HLA-DR expression [mHLA-DR] and ex vivo TNF-alpha release) and HMGB-1 serum levels in patients with severe sepsis/septic shock and sepsis-induced immunosuppression.

## 2. Materials and Methods

### 2.1. Study Population and Drawing of the Samples

36 patients with severe sepsis or septic shock and monocytic deactivation (defined as a monocytic HLA-DR [mHLA-DR] expression <8,000 antigens per cell) were included into the analysis. The analysis presented here was a previously planned subinvestigation of a placebo-controlled trial on the clinical and immunological effects of granulocyte-macrophage colony stimulating factor (GM-CSF) in patients with sepsis and immunoparalysis [22]. After inclusion and randomization, all study patients were attributed to receive either a daily subcutaneous injection of placebo (0.9% NaCl) or GM-CSF (4 *μ*g/kg body weight) for 8 days. 8 *μ*g/kg body weight GM-CSF was given from study days 5 to 8 in two cases of unchanged mHLA-DR expression (mHLA-DR <15,000 antigens per cell at day 5). For assessment of HMGB-1 serum heparinised plasma samples were drawn every other day (at baseline, and study days 3, 5, 7, and 9) from central venous catheters in the morning of each day. All samples were stored at −80 degrees until analysis and samples from all accessible patients entered the analysis. Two patients' samples from the overall analysis [22] were not available and did not enter the analysis. All study patients received intensive care unit (ICU) therapy in adherence to current international guidelines. The study was approved by the local ethics committee on human research (Ethikkomission der Charité Universitätsmedizin Berlin, Germany) and was designed in adherence to the Declaration of Helsinki. Written informed consent was obtained from the patient or respective legal representatives.

### 2.2. Detection of HMGB-1 Serum Levels and Measures of Monocytic Immunity

HMGB-serum levels were assessed using a sandwich ELISA technique (HMGB-1 ELISA kit II, Shino-Test Corporation, Kanagawa, Japan) from 10 *μ*L of heparinised plasma. As stated by the manufacturer, the dynamic range of the HMGB-1 ELISA kit assay was 2.5–82.0 ng/mL. A sensitivity of 1 ng/mL and an intra- and inter assay coefficient of variation <10% applied. Assessment of monocytic function included measurement of ex vivo LPS-induced TNF-*α* release from monocytes (heparinized blood samples, diluted 1 : 10 with RPMI 1640 medium (Biochrom KG, Berlin, Germany), 4 hours of stimulation with 500 pg/mL LPS (Milenia Ex Vivo Whole Blood Stimulation kit, Milenia Biotec, Giessen, Germany) and standardized quantitative determination of the monocytic HLA-DR expression (QuantiBRITE, BD Biosciences, Heidelberg, Germany), as reported elsewhere [22, 26]. Cytokines were determined using the IMMULITE automatic chemiluminescent immunoassay system (Siemens Medical Solutions, Bad Nauheim, Germany). Assessment of respective indices was performed in an accredited (ISO 15189 certified) immunodiagnostic laboratory (Deptartment of Medical Immunology, Charite University Medicine, Berlin, Germany).

### 2.3. Statistical Analysis

All data are presented as mean  ± SD, if not indicated otherwise. Analysis of variance (ANOVA) with Fisher's post hoc test, repeated measures ANOVA, Student's unpaired and paired *t*-tests, simple regression, and chi-square test were used as appropriate. A  *P*-value <.05 was considered significant.

## 3. Results

### 3.1. Study Population

Samples from 36 patients (31 male, aged 64 ± 14, APACHE II score 22 ± 6) with severe sepsis or septic shock were assessed. For detailed patient characteristics, please refer to Table 1 and [22]. Differences in baseline patient characteristics were not noted in regard to the following indices: etiology of sepsis, presence/distribution of gram positive or gram negative/mixed infections, days on the ICU until study inclusion, presence of shock/vasopressor need at baseline, need for renal replacement therapy or mechanical ventilation, and baseline disease severity (APACHE II [27] and SOFA [28] scoring system, n.s. for all comparisons).

### 3.2. Course of HMGB-1 Serum Levels over the 9-Day Intervention Interval

**Figure 1:**
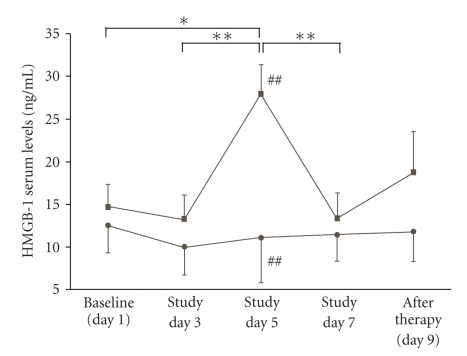
Course of HMGB-1 serum levels (ng/mL) from baseline until study day 9 (after immunotherapy) for GM-CSF (squares) and placebo-treated (circles) individuals. *P ≤ .05, **P ≤ .01 (GM-CSF-treated individuals); ##*P* ≤ .01 between the two groups at the same day of assessment. Means ± SEM are given, paired and unpaired samples *t*-test, as appropriate.

The course of HMGB-1 serum levels (ng/mL) in both study groups is given in Figure 1. In the group receiving immunostimulatory treatment, HMGB-1 serum levels increased significantly until study day 5 (Figure 1), whereas they were unchanged in placebo-treated individuals. A significant between-group difference was identified at study day 5 (27.9 ± 21.7 versus 11.0 ± 14.9 ng/mL, (treatment versus placebo group), *P* = .01). Significant between-group differences were not noted at any other point in time of assessment. From study day 5 until study day 9, HMGB-1 serum levels decreased in the treatment group. Before (baseline) versus after immunotherapy (study day 9) HMGB-1 serum levels were not found to differ in both study groups (both n.s., Figure 1).

### 3.3. Monocytic Immune Function and HMGB-1 Serum Levels

We tested whether HMGB-1 levels correlate with immunostimulation-induced changes in monocytic function. Two aspects of monocytic function were assessed using standardized assays: antigen presentation (i.e., major histocompatibility (MHC) class II surface expression, mHLA-DR) and cytokine (TNF-*α*) release. Although a single immunostimulatory treatment with subcutaneous GM-CSF is known to significantly increase both mHLA-DR expression and TNF-*α* release [22, 23], a consistent long-lasting effect on HMGB-1 serum levels was not noted (Figure 1). Nevertheless, although a short- term increase in HMGB-1 levels was noted at study day 5 (Figure 1), a correlation of both mHLA-DR expression or ex vivo monocytic (LPS-induced) TNF-*α* release with HMGB-1 serum levels was not identified. A correlation between HMGB-1 levels and markers of monocytic function was not noted at any point in time of assessment in any of the study groups (overall study group, treatment group, or placebo group) over the 9-day study interval (all n.s., except mHLA-DR with HMGB-1 at study day 5, *P* = .12; Table 2).

### 3.4. Cytokines and HMGB-1 Serum Levels

Serum levels of mediators which have mostly been referred to as “proinflammatory” (tumor necrosis factor alpha [TNF-*α*], Interleukin [IL]-6), and “anti-inflammatory” (IL-10), as well as procalcitonin (PCT) levels were checked for correlations with HMGB-1 levels in analyses including all samples. Significant correlations between HMGB-1 levels and the aforementioned indices were not identified in the overall, treatment, or placebo groups (all n.s., except TNF-*α* in the subgroup of patients receiving treatment: *P* = .02, *r* = 0.24; data not shown).

### 3.5. Immune Cell Subsets and HMGB-1 Serum Levels

HMGB-1 levels were checked for correlations with the absolute number of leukocytes, natural killer (NK) cells, total number for lymphocytes, B-lymphocytes, T-lymphocytes including CD4 positive and CD8 positive subsets, and monocytes. Significant correlations of HMGB-1 levels with respective indices were identified (Table 3). However, when HMGB-1 levels were adjusted for the total number of leukocytes, the correlation between HMGB-1 levels and markers of monocytic function (mHLA-DR expression, ex vivo TNF-*α* release) and disease severity (APACHE II and SOFA score) remained not significant in the overall samples analysis (all *P* > .29).

### 3.6. Disease Severity (Clinical Scores) and HMGB-1 Serum Levels over Time

We analysed whether HMGB-1 serum levels reflect the course of disease severity in the study population. Therefore, two established clinical scores [27, 28] were investigated whether they correlate with HMGB-1 serum levels before and after immunotherapy (Table 4). HMGB-1 serum levels were not found to correlate with APACHE II and SOFA scores in any study group both at baseline (study day 1) and after therapy (study day 9) (Table 4). These findings were confirmed in an overall samples analysis including samples from both day 1 and day 9 (HMGB-1 versus APACHE II: *P* = .71, *r* = −0.05 [95% CI –0.28–0.19], and HMGB-1 versus SOFA: *P* = .42, *r* = −0.1 [95% CI –0.34–0.15], Table 4). 

## 4. Discussion

Here we demonstrate that immunostimulatory treatment using GM-CSF for sepsis-induced immunosuppression induces a moderate but only transient increase in HMGB-1 levels (Figure 1). Except at study day 5 (after 4 GM-CSF treatments), an association of systemic HMGB-1 levels with indices of monocytic function (mHLA-DR expression, ex vivo TNF-*α* release) was not observed (Table 2). Although we identified moderate correlations between the absolute number of circulating immune cell subsets and HMGB-1 (Table 3), the correlation between HMGB-1 levels and markers of monocytic function were still not significant when the levels of HMGB-1 were adjusted for the number of circulating leukocytes. Moreover, our data indicate that HMGB-1 levels are not associated with disease severity (assessed using the APACHE II and SOFA score, Table 4) and serum levels of both “pro-” (TNF-*α*, IL-6) and “anti-inflammatory” (IL-10) mediators in patients with sepsis-induced immunosuppression. An association between the levels of HMGB-1 and procalcitonin was also not noted. 

Assessment of monocytic activation and monocytic function is recognised a prerequisite for the design and testing of new immunomodulatory therapies in sepsis [17, 19, 20, 22, 25, 26]. Standardized tests for the assessment of monocytic HLA-DR expression and ex vivo LPS-induced monocytic TNF-*α* release have recently been developed and these biomarkers may help to guide immunotherapy for sepsis [23, 26, 29]. In the analysis presented here, we analysed samples from patients with sepsis-induced immunosuppression receiving GM-CSF as a model intervention to reconstruct monocytic immunity. In the past, however, it was debated whether the “late mediator” HMGB-1 reflects monocytic immunity and whether this may guide immunomodulatory interventions in sepsis. Consequently, as increased HMGB-1 serum levels were shown to reflect adverse outcome from sepsis also, targeting of this late proinflammatory mediator was proposed. From our data, however, we conclude that HMGB-1 serum levels do not reflect the course of monocytic immunity in patients with sepsis-induced immunosuppression receiving a specific immunotherapy for this clinical condition. We therefore believe that this parameter should not be used as a primary index for the monitoring of monocytic activation and/or function. This may indeed be a relevant finding for future immunomodulatory interventions. Moreover, we conclude that GM-CSF-induced reversal of monocytic deactivation [22, 23] is not associated with a relevant increase in systemic HMGB-1 levels. 

A number of limitations of our analysis deserve further discussion, among these being the fact that the observational time interval is limited. Thus, the observational period could have simply been too short to notice relevant changes in HMGB-1 serum levels. Although we cannot rule out that this might have affected our findings, we believe that this should not have largely influenced our findings given the fact that GM-CSF obviously induces a moderate but only transient increase in HMGB-1 serum levels. Second, human IgG has been reported to bind to HMGB-1 protein and may interfere with ELISA detection [30]. This might have theoretically influenced our measurements. Third, the scores that we used as surrogate markers for clinical disease severity are not evaluated to assess clinical disease severity in the later course of the disease [27, 28]. Fourth, we demonstrate associations rather than causal relationships in the analysis presented here. Therefore, further ex vivo experiments seem necessary to confirm our findings. In addition, a number of questions remain unanswered, among these the origin of the observed temporary HMGB-1 serum levels increase (Figure 1). As convincingly demonstrated in previous studies, however, HMGB-1 release mostly occurs in response to immune cell activation rather than by passive release following immune cell apoptosis [2–8]. Thus, the observed short-term increase in systemic HMGB-1 levels may have been caused by direct GM-CSF-induced immunostimulatory effects. However, this remains a speculation as the underlying mechanisms need to be elucidated in further studies.

In conclusion, our data demonstrate that HMGB-1 serum levels do not reflect monocytic function in patients with sepsis-induced immunosuppression receiving immunostimulatory treatment. An association of HMGB-1 levels with pro- and anti-inflammatory molecules or clinical disease severity was not observed. We thus believe that HMGB-1 serum levels should not be used as a primary index for the monitoring of monocytic activation or function. This might especially be of importance in subsequent immunomodulatory trials in sepsis.

##  Competing Interests 

All authors declare that they have no competing interests.

## Figures and Tables

**Table 1:** Characterisation of the study cohort.

	control group	treatment group	Between group
	(*n* = 18)	(*n* = 18)	*P*-value
gender (male)	15/18 (83%)	16/18 (89%)	*P* = 1.0
age (years)	64 ± 15	64 ± 14	*P* = .9
body weight (kg)	79 ± 17	82 ± 21	*P* = .6
days on ICU until inclusion	9 ± 9	6 ± 3	*P* = .25
baseline APACHE-II score	22.5 ± 6.9	21.3 ± 6.0	*P* = .6

**Table 2:** Correlation table of systemic HMGB-1 levels with markers of monocytic function.

		HMGB-1 serum level				
		baseline	day 3	day 5	day 7	after therapy
		(day 1)				(day 9)
mHLA-DR expression	- overall group	*P* = .65	*P* = .61	*P* = .12	*P* = .31	*P* = .38
		*r* = 0.08	*r* = 0.09	*r* = 0.27	*r* = 0.18	*r* = 0.16
	- treatment group	*P* = .98	*P* = .90	*P* = .98	*P* = .24	*P* = .73
		*r* = 0.01	*r* = −0.03	*r* = 0.01	*r* = 0.29	*r* = 0.09
	- control group	*P* = .49	*P* = .22	*P* = .97	*P* = .86	*P* = .80
		*r* = 0.18	*r* = 0.31	*r* = 0.01	*r* = −0.05	*r* = 0.07
ex vivo LP-Sinduced TNF-*α* release	- overall group	*P* = .91	*P* = .97	*P* = .77	*P* = .57	*P* = .56
		*r* = −0.02	*r* = −0.01	*r* = 0.05	*r* = −0.10	*r* = 0.11
	- treatment group	*P* = .41	*P* = .50	*P* = .30	*P* = .46	*P* = .69
		*r* = −0.20	*r* = −0.17	*r* = −0.25	*r* = −0.18	*r* = −0.11
	- control group	*P* = .48	*P* = .16	*P* = .97	*P* = .89	*P* = .43
		*r* = 0.18	*r* = 0.36	*r* = −0.01	*r* = −0.03	*r* = 0.21

**Table 3:** Correlation table of systemic HMGB-1 levels with absolute numbers of immune cell subsets.

		HMGB-1 serum level (overall samples analysis)			
		*P*-value	*r* =	95% CI	*n* =
Immune cell subsets	leukocytes	.002	0.24	0.09–0.37	170
	lymphocytes	.001	0.25	0.10–0.39	170
	CD4+ T-lymphoycytes	.012	0.19	0.04–0.33	170
	CD8+ T-lymphoycytes	.025	0.17	0.07–0.35	170
	B-lymphocytes	.036	0.16	0.01–0.30	170
	monocytes	.001	0.25	0.10–0.39	170
	NK cells	.0003	0.27	0.13–0.41	170

**Table 4:** Correlation analysis of systemic HMGB-1 levels with indices of disease severity.

		HMGB-1 serum level		
		baseline (day 1)	after therapy (day 9)	all samples (day 1 + 9)
APACHE II Score	- overall group	*P* = .053	*P* = .32	*P* = .71
		*r* = −0.32	*r* = 0.19	*r* = −0.05
	- treatment group	*P* = .34	*P* = .16	*P* = .34
		*r* = −0.23	*r* = 0.36	*r* = −0.23
	- control group	*P* = .09	*P* = .51	*P* = .09
		*r* = −0.43	*r* = 0.19	*r* = −0.43
SOFA score	- overall group	*P* = .90	*P* = .38	*P* = .42
		*r* = −0.02	*r* = −0.17	*r* = −0.1
	- treatment group	*P* = .58	*P* = .12	*P* = .58
		*r* = −0.14	*r* = −0.39	*r* = 0.14
	- control group	*P* = .82	*P* = .55	*P* = .83
		*r* = −0.06	*r* = −0.17	*r* = −0.06
